# Preparation and Evaluation of Berberine Alginate Beads for Stomach-Specific Delivery

**DOI:** 10.3390/molecules161210347

**Published:** 2011-12-14

**Authors:** Zhen-Hai Zhang, Yong-Shun Sun, Hui Pang, Were L.L. Munyendo, Hui-Xia Lv, Sheng-Liang Zhu

**Affiliations:** 1 Jiangsu Province Academy of Traditional Chinese Medicine, No. 100 shizhijie, Nanjing 210028, China; Email: davidpharm@yeah.net (Z.-H.Z.); 2 Yueyang Hospital of Integrated Traditional Chinese and Western Medicine, Shanghai University of Traditional Chinese Medicine, No. 110 ganhelu, Shanghai 200437, China; Email: yongshunsun@yahoo.com.cn (Y.-S.S.); 3 Jiangsu Institute for Food and Drug Control, No. 6 beijingxilu, Nanjing 210024, China; Email: yupang77@yahoo.com.cn (H.P.); 4 Department of Pharmaceutics, China Pharmaceutical University, No. 24 tongjiaxiang, Nanjing 210009, China

**Keywords:** berberine, octodecanol, calcium alginate beads, buoyancy, gastric residence

## Abstract

The purpose of this research was to prepare floating calcium alginate beads of berberine for targeting the gastric mucosa and prolonging their gastric residence time. The floating beads were prepared by suspending octodecanol and berberine in sodium alginate (SA) solution. The suspension was then dripped into a solution of calcium chloride. The hydrophobic and low-density octodecanol enhanced the sustained-release properties and floating ability of the beads. The bead formulation was optimized for different weight ratios of octodecanol and SA and evaluated in terms of diameter, floating ability and drug loading, entrapment and release. *In vitro* release studies showed that the floating and sustained release time were effectively increased in gastric media by addition of octodecanol. *In vivo* studies with rats showed that a significant increase in gastric residence time of beads had been achieved.

## 1. Introduction

Gastric ulcers are a global health problem with a considerable economic burden associated with its high morbidity and mortality rates [1,2]. Data on the epidemiology, pathophysiology and histology show that *Helicobacter pylori (H. pylori)* infection plays an important role in the development of gastric ulcers and gastric cancer [2,3]. Since its discovery in 1982 by Warren and Marshall and its confirmation as a pathogen in the late 1980s, researchers have attempted in various ways to eradicate *H. pylori* from the stomach. *H. pylori *lives deep inside the gastric mucus layer. Any therapeutic drug must therefore be able to penetrate the gastric mucus layer and maintain a concentration sufficient for antibacterial activity at the infected site for a suitable length of time [4,5]. Several approaches have been pursued to increase the retention of an oral dosage form in the stomach, including high-density systems [6], nanoparticles [7], bioadhesive systems [8], superporous hydrogels [9] and floating delivery systems [10].

Floating drug delivery systems are designed with low bulk density to remain buoyant in the stomach for a prolonged period of time without being affected by the gastric emptying rate. While the system is floating on the gastric contents, the drug is slowly released at a desired rate. Floating drug delivery systems have the advantage of allowing local delivery of a drug to the stomach. They also reduce the variability in bioavailability that is encountered with some current immediate and modified release systems [11]. Lately, both natural and synthetic hydrophilic polyionic systems like alginates have been investigated for preparation of floating systems. However various floating alginate beads suffered from rapid drug release [12,13,14].

Sodium alginate (SA) has been used to treat the symptoms of reflux esophagitis [15,16]; and the result showed that the sodium alginate was more effective, and costs less, than cisapride for the treatment of symptoms presented by patients suffering from reflux without severe oesophagitis. Literature reports have indicated widespread use of sodium alginate for achieving sustained release of drugs, targeting gastric mucosa [17], and increasing the bioavailability of drugs [18], due to the ability of sodium alginate to form a stable and bioadhesive gel with calcium ions [19]. In addition, the alginate bead preparation method involves the use of aqueous solvents, avoiding exposure of ingredients to high temperatures and toxic organic solvents. Moreover, the resulting preparation is non-immunogenic, with bioadhesive and floating properties that could be appropriate for stomach targeted drug delivery.

Berberine is a natural isoquinoline quaternary alkaloid derived from a number of species of the barberry plant, including *Berberis aristate* and *Coptis chinensis*, which has been widely used in Chinese herbal medicine. Berberine has multiple biochemical and pharmacological effects, including anticancer, antiviral, and antibacterial activities [20,21,22]. Recent pharmacological studies have demonstrated that berberine is able to exert inhibitory effects on the proliferation capacity of *H. pylori* and activities of *H. pyloriN*-acetyltransferase [23].

The floating delivery system strategy allows local delivery of a drug to the stomach, making it a promising vehicle for berberine. The work described here is concerned with the formulation of berberine floating calcium alginate beads. Such a dosage form for berberine would remain buoyant in the stomach without effecting the gastric emptying time for a prolonged period of time [24] and localize drug at the *H. pylori* infection site on the gastric epithelium. Furthermore, such treatment may lead to drug dose reduction which will be an additional valuable advantage [25].

In this study floating alginate beads composed of berberine and octodecanol are prepared. Mean diameter, drug loading, drug entrapment, floating ability, drug release and gastric retention time in rats were evaluated as desirable parameters.

## 2. Results and Discussion

### 2.1. Preparation and Evaluation of Floating Berberine Beads

The size and sustained release characteristics of berberin calcium alginate beads were found to be influenced by the composition formulae and process parameters [26,27]. After many preliminary trials it was observed that viscosity and the concentration of SA; concentration of CaCl_2_ the inner diameter of needle; dripping height from needle, and the proportion of drug and adjunctions were all important.

The most important factor considered was viscosity and the concentration of SA. In the study up to seven types of sodium alginate with different viscosities ranging from 50 cps~1,300 cps were used. The result showed that the viscosity of SA affected the spheroid shape of the beads. Use of LVCR (50 cps viscosity) resulted in beads observed to be shrinking, while the beads prepared with Kecosol (1,300 cps) displayed tailing. Suitable spheroid shaped beads were obtained only when SA of viscosity in the range 220 cps to 500 cps was used. The beads were perfect spheroids when the concentration of SA solution was in the range of 1~2%.

The inner diameter of syringe needle was also an important factor influencing the size of beads. Four needles of 4, 6, 8 and 12 mm inner diameter gave free beads of 0.75 mm, 0.86 mm, 0.97 mm and 1.13 mm mean diameter, respectively. There was no significant difference in floating ability among the four sizes of beads. Sustained-release increased with increase in the diameter of beads, so the needle of 12 mm inner diameter was therefore selected.

A more than 2% concentration of CaCl_2_ was necessary for beads that could resist being crushed by liquid droplets. Suitable spheroid shaped beads were achieved while dripping from a height of 5~8 cm. The beads aggregated when the height was less than 5 cm, and could be flat sphere at more than 8 cm.

**Figure 1:**
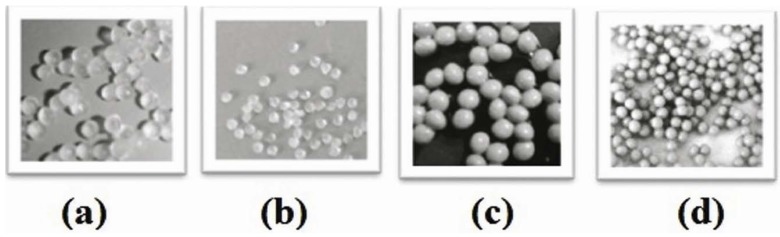
Calcium alginate beads prepared by the dripping technique (the four figures are not at the same ratio); (**a**) the free beads before dehydration (mean diameter = 2.44 mm); (**b**) the free beads after dehydration, (mean diameter = 0.85 mm); (**c**) the berberine beads before dehydration with 88.7% drug loading (mean diameter = 2.98 mm); (**d**) the berberine beads after dehydration with 88.7% drug loading (mean diameter = 2.07 mm).

The size of SA beads was changed largely after drying [Figure 1(a) and (b)]. The mean diameter of free beads before and after drying was 2.44 mm and 0.85 mm, respectively (2.9-fold change). Meanwhile an increase in the proportion of berberine (1:2 to 1:10) led to a decrease in the size changing tendency (experimental details are not given due to space limitations). At 88.7% drug loading, the mean diameter of beads before and after drying was 2.98 mm and 2.078 mm, respectively [1.4-fold change, Figure 1(c) and (d)].

#### 2.1.1. Diameter

The mean particle size of the six formulations ranged from 0.94 ± 0.01 to 2.07 ± 0.01 mm. It was observed that an increase in the proportion of berberine (0:1 to 1:10) led to an increase in the size of beads (Table 1). This indicated that the ratio of drug also influenced the size of beads, revealing that the change in size of the calcium alginate beads also depends on the added material, and not only on the process parameters. The standard deviations indicate that individual variability was low.

**Table 1:** The characterization of beads in different ratios SA to berberine (n > 100).

Ratios (SA: berberine)	1:0	1:2	1:4	1:6	1:8	1:10
Mean Diameter (mm)	0.85 ± 0.02	1.24 ± 0.01	1.42 ± 0.01	1.69 ± 0.01	1.86 ± 0.01	2.07 ± 0.01
Drug Loading (%)	0	62.1	75.5	81.4	85.4	88.7
Entrapped Efficiency (%)	0	94.6	94.4	94.7	91.3	95.1

Note: The six formulations contained 6.25% of octodecanol (wt/wt).

#### 2.1.2. Drug Loading and Drug Entrapment Efficiency

The percent drug loading of the various formulations was from 62.1% to 88.7%, while entrapment efficiency was found to vary between 91.3% and 95.1% (Table 1). It was clearly shown that there was no significant difference in drug entrapment efficiency for the five formulas, which might be due to the saturated berberine solution of CaCl_2_.

#### 2.1.3. Floating Ability

The floating ability of the prepared beads containing berberine was evaluated alongside dissolution studies (Table 2). Beads containing octodecanol demonstrated instantaneous and excellent floating ability, while beads without octodecanol sank immediately in HCl buffer (pH 1.2) but were observed to be suspended at the centre of the dissolution vessels after 1 h. The floating ability was found to be directly related to the content of the wax matrix. At octodecanol concentrations above 6.25%, all the beads floated immediately upon contact with the release medium and maintained buoyancy for more than 24 h. This result proved that octodecanol was an ideal adjuvant as a light wax matrix providing a low total system density, which has been confirmed by several researchers [28,29].

On lyophilization, the beads of F5 could be dehydrated forming a porous system that floats after 5 min and remained floating for over 20 h. Although the beads had a floating time of more than 12 h, there exists a problem whereby the entire drug was realesed within 3 h. So the method of bead drying in oven was chose.

**Table 2:** Lag time and lasting floating time of different formulas (n = 6).

No.	Ratio (SA:octodecanol:bererine)	Octodecanol (%)	Lag time (h)	Lasting floating time (h)
F1	1:1:2	25%	0	>24
F2	1:0.5:2	14.29%	0	>24
F3	1:0.2:2	6.25%	0	>24
F4	1:0.1:2	3.23%	0.1 *	15.3
F5	1:0:2	0	sank immediately and then suspended in the central of vessels after 1 h	

* Lag time = time from the introduction to when all beads floated on the test solution.

### 2.2. *In vitro* Dissolution Studies

**Figure 2:**
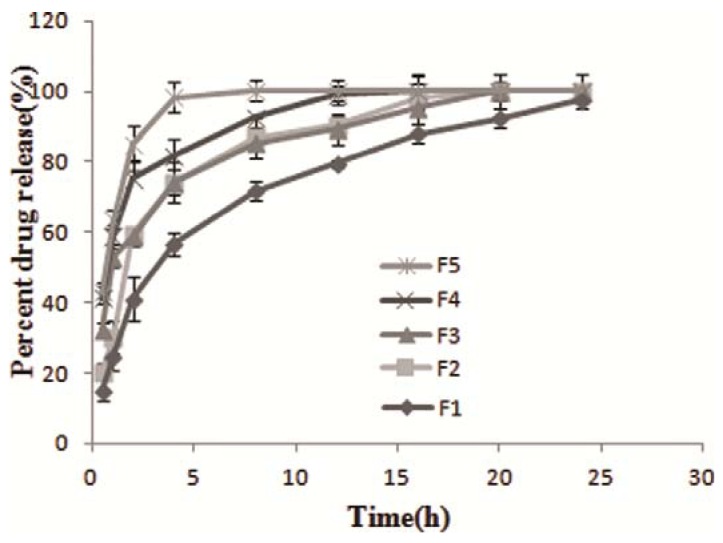
Release profile from berbeine SA beads.

Formula 1 (F1) was found to be best in the *in vitro* drug release studies, with up to 100% release after 24 h (Figure 2) and a floating time of more than 24 h (Table 2). The other formulas (F2 to F4) showed faster release than F1. It could therefore be concluded that octodecanol was effective to enhance sustained drug release while prolonging floating time of the beads. However with increasing the percentage of octodecanol, the berberine beads were more difficult to prepare and showed tailing of their spheroid shape.

The model that gives high “r^2^” value is considered as the best fit of the release data. Drug transport inside pharmaceutical systems involves multiple steps provoked by different physical or chemical phenomenon, making it difficult, or even impossible, to get a mathematical model describing it in the correct way. In general the release models with major application and best describing drug release phenomena are zero order model, first order model the Higuchi model, Hixson-Crowell and Korsemeyer-Peppas model [30]. To evaluate the mechanism of berberine release from F1 beads profile, the results of regression for the above models were shown in Table 3. The highest “r^2^” values of 0.9973 and 0.9968 indicated the berberine release model was considered as first order and Korsemeyer-Peppas mechanism.

**Table 3:** Regression result of SA release from stomach retention gel beads.

No	Model	Results	r^2^
1	Zero order	Q_t_ = 5.1061t	0.3365
2	Fist order	Q_t_ = 21.707Lnt + 26.995	0.9973
3	Higuich	Q_t_ = 21.595t^1/2^	0.9362
4	Hixson-Crowell	(100 − Q_t_)^1/3^ = −0.1208t + 4.2502	0.9860
5	Korsemeyer-Peppas	LnQ_t_ = 0.3837Lnt + 2.8229	0.9968

Note: where r^2^ was Correlation coefficient, Q_t_ was cumulative amount of drug release at time “t”.

For the spheroids matrix perparations, an exponent n ≤ 0.45, indicates a Fickian diffusion drug release mechanism (dM = K△C/dt), and if 0.45 < n < 0.89, then a non-Fickian or anomalous diffusion. The exponent value of F1 (0.3837) obtained is thus indicative of Fickian diffusion release. For Fickian diffusion, the value of K was related to the surface of sphere parameters; diffusion coefficient and solubility of drug. If these parameters were constant, the mechanism of drug release accorded with zero order, compared with non-zero level for the variable parameters [30].

The calcium alginate formed alginic acid since calcium ion of calcium alginate beads could be exchanged by hydrogen ion of HCl buffer (pH 1.2). Alginic acid is insoluble on inflation, and it was therefore suggested that the release mechanism of bererine in calcium alginate beads was pore diffusion from an insoluble polymer matrix and in accordance with first order or Korsemeyer-Peppas model.

### 2.3. *In Vivo* Gastric Residence Time Study in Rat

Beads retention in the stomach (Table 4) showed that gastric retention increased from at concentration of octodecanol above 6.25%.

**Table 4:** Results of gel beads stomach retention ability in rat.

Formula	Remaining bead (12 h)	Remaining bead (24 h)
berberine alginate beads 1	30	30
berberine alginate beads 2	30	29
berberine alginate beads 3	27	22
free alginate beads	22	15
sustained-release micro-pellets	12	6

Note: The SA:octodecanol:berberine ratios for the different formulas were as follows: berberine alginate beads 1 = 1:1:2 berberine alginate beads 2 = 1:0.5:2; berberine alginate beads 3 = 1:0.2:2; The remained beads were the sum total of 3 rats.

Compared with marketed sustained-release pellets, the free SA beads displayed a better retention performance in stomach. This remarkable gastric residence time involves the gastric muco-adhesion ability of sodium alginate [19], and without being affected by the gastric emptying time [24].

## 3. Experimental

### 3.1. Materials

Berberine and octodecanol (stearyl alcohol) was purchased from Nanjing Chemical Reagent Factory China. Samples of SA Nos. 1~3 (1, viscosity: 220 cps; 2, viscosity: 430 cps; 3, viscosity: 530 cps) were a kind gift from China Bright Moon Seaweed Group. SA samples Nos. 4~7 (LVCR, viscosity: 50 cps; DMB, viscosity: 300 cps; HVCR, viscosity: 400 cps; Kecosol, viscosity: 1,300 cps) were a present from UK ISP Alginate Ltd. Diclofenac sodium sustained release micro-pellets were purchased from Nanjing Drum Tower Hospital. All the other chemicals were of analytical grade and used without further purification.

### 3.2. Preparation and Evaluation of Floating Berberine Beads

SA was dissolved in the distilled water. Octodecanol was melted at 50 °C and added to berberine powder. The mixture of bererine and octodecanol was then added to the SA solution. The resulting suspension was dropped through a 12 mm inner diameter syringe needle from a height of 7 cm into calcium chloride solution (2% w/v, 200 mL) saturated with berberine. The formed beads were left in the same solution for 45 min to improve their mechanical strength and finally dried at 50 °C in an oven.

#### 3.2.1. Determination of Mean Diameter

The prepared beads (n > 100) was lined and diameter determined by Vernier caliper. Measurements for each sample were performed in triplicate. Mean diameter and its standard deviations were recorded.

#### 3.2.2. Determination of Drug Loading and Drug Entrapment Efficiency

The prepared beads were evaluated for percent drug loading and drug entrapment efficiency. An accurately weighed sample of beads was crushed in a mortar and dissolved in pH 1.2 HCl solution (100 mL). This mixture was then centrifuged at 4,200 rpm for 30 min and filtered using 0.22 μm microporous membrane before analysis with a UV spectrophotomer at 346 nm. The percent drug loading (DL) and drug entrapment efficiency (EE) were calculated using the following Equations (1) and (2):

DL = weight _berberine_ / weight _bead_ (1)

EE％ = weight_ berberine 1_ / weight_ berberine 2_ (2)

where the weight _berberine 1_ was the amount of berberine in all berberine alginate beads and weight _berberine 2_ was the initial berberine amount added into SA solution.

#### 3.2.3. Determination of Floating Time

The floating ability was determined using China Pharmacopeia XC dissolution tester apparatus II (paddle method). Fifty beads were introduced in the vessels and the paddles rotated at 50 rpm in pH 1.2 HCl solution (500 mL), maintained at 37 ± 0.5 °C. The floating ability of the beads was measured by visual observation and the percent of floating beads recorded as average of three determinations. The preparation was considered to have buoyancy only when all beads floated on the test solution immediately or within a lag time which did not exceed 2 min.

### 3.3. *In Vitro* Dissolution Study

*In vitro* dissolution studies were performed for all the formulation combinations in hexaplicate using a China Pharmacopeia XC dissolution tester apparatus II (paddle method). An accurately weighed sample of berberine alginate beads was dropped into pH 1.2 HCl solution (900 mL) maintained at a temperature of 37 °C ± 0.5 °C at 50 rpm of paddle stirring. At different time intervals, an aliquot of the sample (10 mL) was withdrawn and filtered. The filtrate was analyzed at 346 nm using a UV spectrophotometer against pH 1.2 HCl solution as blank.

### 3.4. *In Vitro* Gastric Residence Time Study in Rat

Thirty male Sprague-Dawley rats were used (210 ± 10 g body weight) and treated according to the regulations for animal care of China Pharmaceutical University. The rats were allowed free access to water but were fasted for 12 h before administration. The animals were randomly divided to five groups. Each group was administrated free alginate beads, berberine alginate beads and diclofenac sodium sustained release micro-pellets coated with ethyl cellulose, respectively (10 beads or pellets per rats). The rats were allowed free access to water and then dissected 12 and 24 h after administration. The remaining piece of beads and pellets in the stomach were observed.

## 4. Conclusions

In this study, we prepared floating alginate bead of berberine together with octodecanol and evaluated different parameters including diameter, drug loading, drug entrapment, floating ability and drug release. These parameters are applicable not only to the sustained release of drugs, but also to the targeting of the gastric mucosa. The formulation F1 exhibited the optimum sustained release of berberine, with excellent floating characteristics. Therefore, the alginate beads containing octodecanol appears to be a promising vehicle for drug delivery to the gastric mucosa in the therapy of *H. pylori* infection.

## Supplementary Materials

Supplementary File 1
