# Supplementary figures and images for: Preparation and Evaluation of Berberine Alginate Beads for Stomach-Specific Delivery
Source: Molecules. 2011 Dec 14;16(12):10347–56. doi: 10.3390/molecules161210347 (PMC6264251; doi:10.3390/molecules161210347)

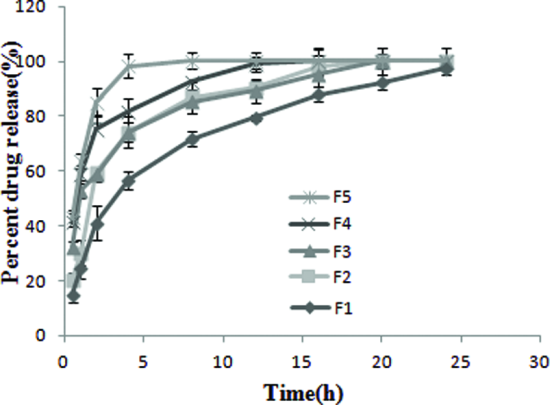

Supplement: Supplementary File 1 [file molecules-16-10347-s001.png]
